# Altered kynurenine pathway metabolites in serum of chronic migraine patients

**DOI:** 10.1186/s10194-016-0638-5

**Published:** 2016-04-29

**Authors:** Martina Curto, Luana Lionetto, Andrea Negro, Matilde Capi, Francesco Fazio, Maria Adele Giamberardino, Maurizio Simmaco, Ferdinando Nicoletti, Paolo Martelletti

**Affiliations:** Department of Psychiatry, Harvard Medical School, Boston, MA USA; Department of Molecular Medicine, Sant’Andrea Medical Center, Sapienza University, Via di Grottarossa 1035-1039, Rome, 00189 Italy; Advanced Molecular Diagnostics, IDI-IRCSS, Rome, Italy; Regional referral headache center, Sant’Andrea Hospital, Rome, Italy; IRCCS Neuromed, Pozzilli, Italy; Headache Center and Geriatrics Clinic, Department of Medicine and Science of Aging, “G. D’Annunzio” University, Chieti, Italy; Department of Physiology and Pharmacology, Sapienza University, Rome, Italy

**Keywords:** Chronic migraine, Glutamate, Kynurenine, Metabotropic Glu receptors, NMDA receptors, Pain

## Abstract

**Background:**

Activation of glutamate (Glu) receptors plays a key role in the pathophysiology of migraine. Both NMDA and metabotropic Glu receptors are activated or inhibited by metabolites of the kynurenine pathway, such as kynureninic acid (KYNA), quinolinic acid (QUINA), and xanthurenic acid (XA). In spite of the extensive research carried out on KYNA and other kynurenine metabolites in experimental models of migraine, no studies have ever been carried out in humans. Here, we measured all metabolites of the kynurenine pathway in the serum of patients affected by chronic migraine (CM) and age- and gender-matched healthy controls.

**Methods:**

We assessed serum levels of tryptophan (Trp), L-kynurenine (KYN), KYNA, anthranilic acid (ANA), 3-hydroxyanthranilic acid (3-HANA), 3-hydroxykynirenine (3-HK), XA, QUINA, and 5-hydroxyindolacetic acid (5-HIAA) in 119 patients affected by CM (ICHD-3beta criteria) and 84 age-matched healthy subjects.

Patients with psychiatric co-morbidities, systemic inflammatory, endocrine or neurological disorders, and mental retardation were excluded. Serum levels of all metabolites were assayed using liquid chromatography/tandem mass spectrometry (LC-MS/MS).

**Results:**

LC-MS/MS analysis of kynurenine metabolites showed significant reductions in the levels of KYN (−32 %), KYNA (−25 %), 3-HK (−49 %), 3-HANA (−63 %), 5-HIAA (−36 %) and QUINA (−80 %) in the serum of the CM patients, as compared to healthy controls. Conversely, levels of Trp, ANA and XA were significantly increased in CM patients (+5 %, +339 % and +28 %, respectively).

**Conclusions:**

These findings suggest that in migraine KYN is unidirectionally metabolized into ANA at expenses of KYNA and 3-HK. The reduction in the levels of KYNA, which behaves as a competitive antagonist of the glycine site of NMDA receptors, is consistent with the hypothesis that NMDA receptors are overactive in migraine. The increase in XA, a putative activator of Glu2 receptors, may represent a compensatory event aimed at reinforcing endogenous analgesic mechanisms. The large increase in the levels of ANA encourages research aimed at establishing whether ANA has any role in the regulation of nociceptive transmission.

## Background

Among primary headache disorders, migraine is common in clinical practice [1]. It is characterized by a natural fluctuation between a low and a high frequency pattern under the influence of modifiable and non-modifiable risk factors [2]. Increased attack frequency may lead to the so-called ‘chronic migraine’ (CM), which becomes less responsive to both acute and prophylactic treatments. CM is associated with significant disability and high socioeconomic impact [3, 4], which make CM a major public health problem [5, 6]. In spite of the increasing number of drugs marketed for the treatment of migraine, a substantial proportion of patients do not respond to medication, and are therefore at risk for chronicization [7].

**Fig. 1:**
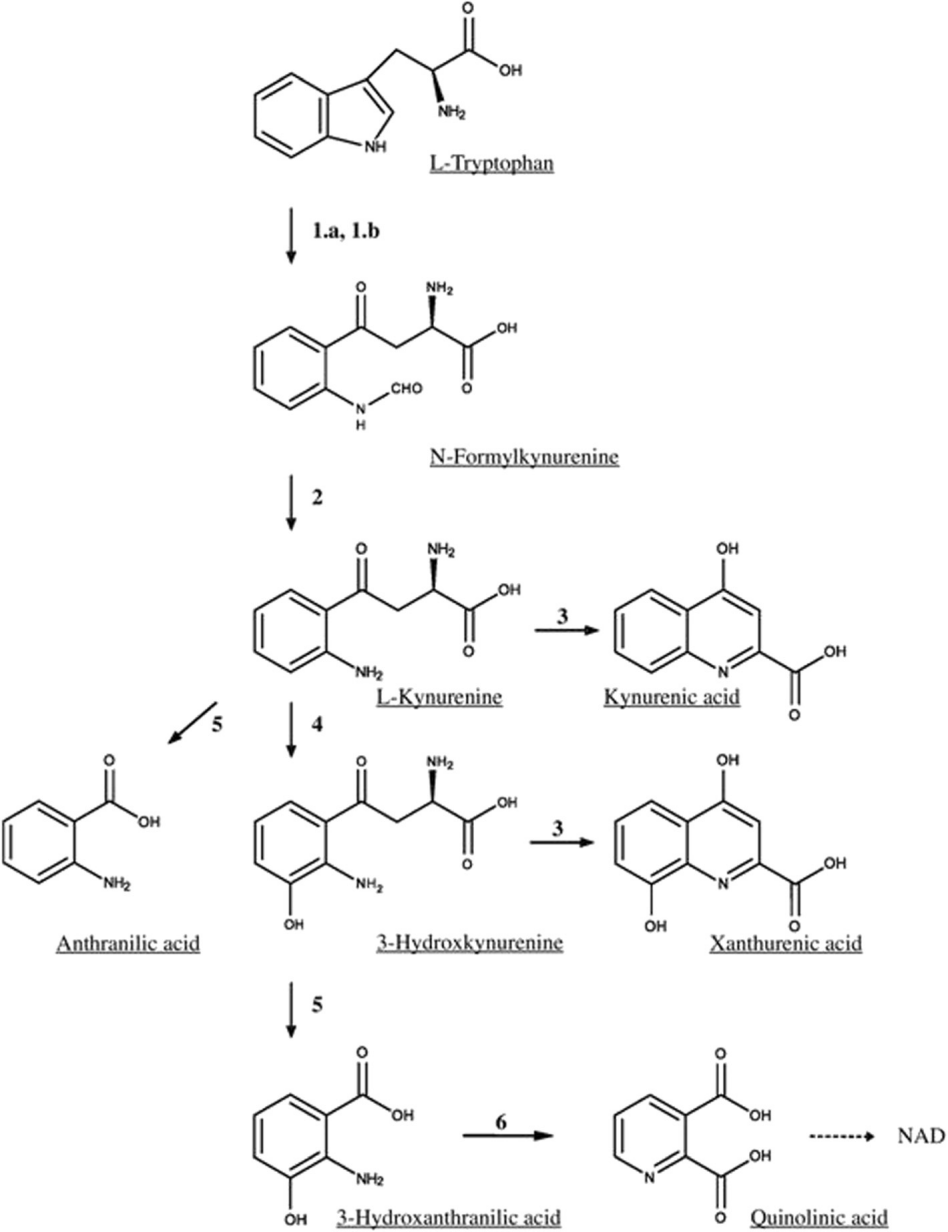
The Kynurenine Pathway of Tryptophan metabolism. The kynurenine pathway. (*1.a*) Indoleamine-pyrrole 2,3-dioxygenase; (*1.b*) tryptophan 2,3-dioxygenase; (2) arylamine formamidase; (3) kynurenine aminotransferase; (4) kynurenine 3-hydroxylase; (5) kynureninase; (6) 3-hydroxyanthranilic acid 3,4-dioxygenase

Four not-mutually-exclusive mechanisms have been implicated in the pathophysiology of migraine: (i) peripheral sensitization of the trigeminovascular system; (ii) central sensitization of the caudal trigeminal nucleus and other central nervous system (CNS) structures of the pain neuraxis; (iii) activation of brainstem migraine generators; and, (iv) cortical spreading depression, underlying aura phenomenon of migraine with aura [8]. All these processes are mediated by allostatic changes in mechanisms of activity-dependent synaptic plasticity, such as long-term potentiation (LTP) and long-term depression (LTD). Glutamate (Glu), the major excitatory neurotransmitter, is involved in the induction and expression of both LTP and LTD, and, therefore, extensive research has focused on the role of Glutamatergic transmission in the pathophysiology of migraine. N-methyl-D-aspartate (NMDA) receptors, have an established role in mechanisms of central sensitization [9, 10] and in the onset and propagation of cortical spreading depression [11, 12]; in addition, an association has been found between migraine and polymorphisms in genes involved in the regulation of synaptic Glu uptake and postsynaptic Glu signaling [13–15]. Increased plasma and cerebrospinal fluid Glu levels have been reported in patients affected by migraine [16, 17], but the interpretation of these findings is limited by the large prevalence of the metabolic pool of Glu with respect to the neurotransmitter pool. Investigation on other endogenous ligands of Glu receptors in patients affected by migraine is limited. The KP of Trp metabolism generates a series of compounds that interact with Glu receptors, and, therefore, have been implicated in the pathophysiology of migraine [18]. The pathway is activated by either indolamine 2,3-dioxygenase (IDO) or tryptophan 2,3-dioxygenase (TDO), which transform L-tryptophan into N-formylkynurenine. L-kynurenine (KYN), 3-hydroxykynurenine (3-HK), kynurenic acid (KYNA) anthranilic acid (ANA), xanthurenic acid (XA), 3-hydroxyanthranylic acid (3-HANA) and quinolinic acid (QUINA) synthesis are shown in Fig. 1. KYNA inhibits NMDA receptors behaving as a competitive antagonist at the glycine site of the GluN1 subunit, whereas QUINA is an orthosteric agonist at the GluN2A-D subunits of NMDA receptors [19]. Recent evidence suggests that XA behaves as an endogenous activator of type-2 metabotropic Glu receptors (mGlu2 receptors) [20], whereas cinnabarinic acid is a weak orthosteric agonist of mGlu4 receptors [21].

The influence of peripherally administered KYNA or its precursor, KYN, on the trigeminovascular system or cortical spreading depression has been the subject of extensive investigation, and pharmacological strategies aimed at enhancing KYNA synthesis have been suggested in migraine [8] (see also Discussion and References therein).

To our knowledge, no measurements of kynurenine metabolites have been performed in the plasma of patients affected by migraine or other forms of chronic headache. This aspect is not trivial because brain levels of IDO and TDO are low, and peripheral L-kynurenine and 3-HK fuel the kynurenine pathway in the CNS [19]. Here we report the surprising finding that ANA levels are substantially increased in the plasma of patients affected by CM, at the expenses of all other kynurenine metabolites with the notable exception of XA.

## Methods

### Patients

The protocol was carried out in accordance with the declaration of Helsinki and the study design was reviewed and approved by the Ethical Committee at Sapienza, University of Rome, Sant’Andrea Hospital. All subjects signed free informed consent for participation in the study. All subjects were enrolled by the Regional Referral Headache Center of S. Andrea Hospital and evaluated by two experts in headache disorders (A.N. and P.M.). 119 patients met the ICHD-3beta criteria for CM and were included in the CM group, and 84 healthy subjects were included in the age-matched control group. Inclusion criteria for CM patients were: (i) age between 18 and 75 years; (ii) patients treated with OnabotulinumtoxinA for the prophylactic therapy (iii) patients treated with triptans or any other analgesics during the headache attacks.

Exclusion criteria were: (i) the presence of psychiatric co-morbidities, immunological disorders, endocrine disorders, neurological disorders, and mental retardation; (ii) lifetime history of migraine (for healthy volunteers); and (iii) the use of any drug of abuse in the last 3 months (except cigarette smoking). Both patients in an attack free and in a pain period were included.

### Blood collection and KP measurement

Serum samples from patients and controls were collected between 10 am and 12 am. Blood (5 ml) was sampled in anticoagulant-free tubes and kept at room temperature for 1 h before the serum was isolated (centrifugation at 2000 g for 10 min at 20 °C).

We developed a liquid chromatography/tandem mass spectrometry (LC-MS/MS) method for the assay of serum levels of all kynurenine metabolites. The method allowed a reliable detection of KYN, KYNA, ANA, 3-HANA, 3-HK, XA, and QUINA. Levels of Trp and 5-HIAA were also detected. Details on sample preparation, reagents, standard solutions, chromatographic conditions, mass spectrometry conditions, and validation parameters are reported in Fazio et al., [20].

### Statistical analysis

SPSS version 19.00 (IBM Corporation, Armonk NY, USA) was used for data analysis. Continuous variables were expressed as mean standard ± deviation (SD), while discrete variables were assigned as numbers and percentages. The compliance of continuous variables with normal distribution was controlled with the Kolmogorov–Smirnov test. Whether Healthy Controls and Cluster Headache groups differed in terms of discrete variables was checked through Pearson's Chi Squared test (*χ*2). Since continuous variables did not comply with a normal distribution, the Mann–Whitney *U* test was used for between group comparisons. We set statistical significance at *P* ≤ 0.05.

## Results

Demographic characteristics of patients affected by CM (*n* = 119) and age-matched healthy controls (HCs) (*n* = 84) are shown in Table 1. The CM group, as expected, showed a prevalence of female gender (72.3 %), which did not differ from the percentage of female gender in the selected HCs (82.1 %). Mean age was 44 and 40 years in patients affected by CM and in HCs, respectively (see Table 1).

**Table 1:** Serum levels of kynurenine metabolites, tryptophan, and 5-HIAA, in patients affected by chronic migraine (CM) and healthy controls

	Healthy controls	Patients with CM	χ^2^/U
	(*n* = 84)	(*n* = 119)	*p*
Gender (F, %)	69 (82.1)	86 (72.3)	2.66 0.071
Age (years; mean ± SD)	40.4 ± 9.43	44.0 ± 11.6	4446 0.180
Trp (μg/ml) (mean ± SD)	5.00 ± 1.55	5.27 ± 1.70	4198 0.052
KYN (μg/ml) (mean ± SD)	0.34 ± 0.12	0.23 ± 0.09	2286 <0.001
KYNA (ng/ml) (mean ± SD)	3.27 ± 1.85	2.46 ± 1.29	3692 0.002
ANA (ng/ml) (mean ± SD)	2.28 ± 2.14	10.0 ± 5.92	565 <0.001
3-HK (ng/ml) (mean ± SD)	2.39 ± 3.14	1.21 ± 0.69	3498 <0.001
3-HANA (ng/ml) (mean ± SD)	9.11 ± 4.33	3.42 ± 1.40	553 <0.001
QUINA (ng/ml) (mean ± SD)	18.2 ± 12.5	3.78 ± 3.34	580 <0.001
5-HIAA (ng/ml) (mean ± SD)	32.6 ± 11.4	20.8 ± 8.16	1991 <0.001
XA (ng/ml) (mean ± SD)	1.80 ± 1.01	2.30 ± 1.52	4108 0.048

*Trp* tryptophan, *KYN* L-kynurenine, *KYNA* kynurenic acid, *ANA* anthranilic acid, *3-HK* 3-hydroxykynurenine, *3 – HANA* 3-hydroxyanthranylic acid, *QUINA* quinolinic acid, *5-HIAA* 5-hydroxyindoleacetic acid, *XA* xanthurenic acid (XA)

**Fig. 2:**
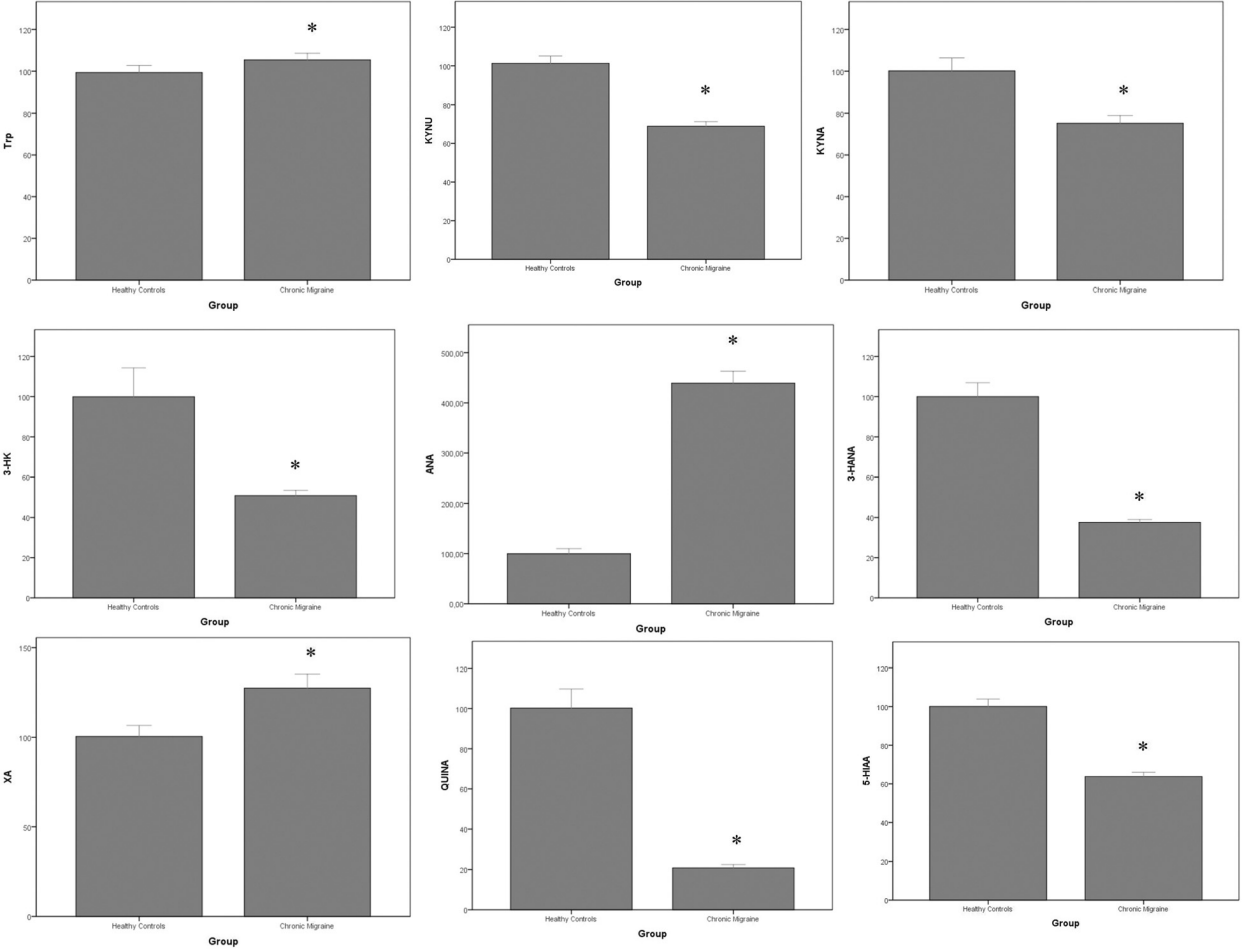
Percent changes in serum kynurenine metabolites in patients affected by CM with respect to healthy controls. Data are presented as per cent of HC values for each metabolite. Values are means ± S.E.M. *p < 0.05 vs. healthy controls (HCs)

LC-MS/MS analysis of kynurenine metabolites showed significant reductions in serum levels of KYN (−32 %), KYNA (−25 %), 3-HK (−49 %), 3-HANA (−63 %), and QUINA (−80 %). Surprisingly, ANA levels were largely increased (+339 %) in the serum of patients affected by CM. XA levels were also increased in CM patients, but to a lower extent (+28 %). We extended the analysis to the precursor L-Trp, and the serotonin metabolite, 5-HIAA. We found a small, but significant, increase in Trp levels (+5 %) associated with a substantial reduction in 5-HIAA levels in the serum of patients affected by CM (Table 1, Fig. 2). No difference between serum levels of any kynurenine metabolites was found between male and female subjects in CM patients and HCs (not shown).

## Discussion

We have shown for the first time that CM is associated with abnormalities of the kynurenine pathway, as reflected by changes in serum levels of KYN and all downstream metabolites. An important question in all studies in which putative neuroactive molecules are measured in peripheral blood is whether, and to what extent, blood levels reflect CNS levels. It has been demonstrated that peripheral KYN and 3-HK cross the blood–brain barrier and fuel the KP in the CNS, where IDO and TDO activities are constitutively low. Brain penetration is lower for other kynurenine metabolites (reviewed by Schwarcz et al., [19]). Thus, we cannot exclude that changes of at least some kynurenine metabolites we have found in CM patients originate in the liver and other organs rather than in the CNS. In spite of these limitations, our data indicate that CM is associated with substantial changes in the levels of kynurenine metabolites that are not easy to explain on the basis of our classical knowledge of the KP. In CM patients, levels of all metabolites that lie downstream of KYN were largely reduced, with the exception of ANA levels, which were dramatically increased, and XA levels, which were increased to a much lower extent. These findings suggest that KYN is rapidly and unidirectionally metabolized into ANA at expenses of KYNA and 3-HK. This is unusual, and we are not aware of any other conditions in which a large accumulation of ANA is associated with a reduction of most of all other kynurenine metabolites. The large accumulation of ANA suggests that its transformation into 3-HANA is largely reduced in migraine. The significant increase in XA levels found in CM patients is also difficult to explain because the enzyme Kynurenine Aminotrasferase (KAT), which transaminates 3-HK into XA also converts KYN into KYNA (reviewed by Schwarcz et al., [19]), the levels of which were reduced in CM patients. Perhaps the degradation of XA is defective in migraine. However, this hypothesis could not be tested because of the incomplete knowledge of XA catabolism.

We will attempt to correlate these changes to the pathophysiology of migraine to the best of our knowledge. The study of kynurenine metabolites in models of migraine has been focused on KYNA, which displays pleiotropic actions in the CNS by inhibiting NMDA receptors and α7-nicotinic acetylcholine receptors, and activating the GPR35 G-protein coupled receptor [22]. KAT, the enzyme that transforms KYN into KYNA, is found in Schwann cells, macrophages, and mast cells in the supratentorial dura mater of rats, and its activity is reduced by stimulation of the trigeminal ganglion [23]. KYN (boosted with probenecid) or synthetic analogues of KYNA given systemically inhibit the activation of the caudal trigeminal nucleus in experimental animal models of migraine [24–28]. KYN and KYNA also inhibit the development of cortical spreading depression and the associated changes in the permeability of the blood brain barrier, and at least the effect of KYN is modulated by sex hormones [22, 29, 30]. Taken together, these findings suggest that KYNA has a protective role against migraine by restraining the activation of NMDA receptors. The reduction in KYNA levels we have seen in the serum of CM patients is consistent with the hypothesis that NMDA receptors are overactive in migraine (see Background and References therein).

Interestingly, CSF and serum KYNA levels are increased in patients affected by schizophrenia [19, 20], a CNS disorder characterized by a hypofunction of NMDA receptors [31]. Thus, peripheral KYNA levels might represent a reliable indicator of NMDA receptor function in distinct CNS disorders.

One might argue that the large reduction in serum QUINA levels we have seen in CM patients may limit the activation of NMDA receptors in migraine because QUINA behaves as an orthosteric NMDA receptor agonist [32]. However, migraine is characterized by an increased glutamatergic transmission [8, 11], and, under these conditions, NMDA receptors may be fully activated by the high synaptic Glu levels combined with low KYNA levels. Thus, at least in migraine, the reduction in KYNA levels might facilitate NMDA receptor activation in spite of the simultaneous reduction in QUINA levels.

Recent findings suggest that XA activates mGlu2 and mGlu3 receptors [20], although XA may also indirectly influence glutamatergic transmission by interacting with vesicular Glu transporters [33, 34]. mGlu2 receptors negatively modulate pain transmission, and drugs that activate or induce mGlu2 receptors are promising candidates in the experimental treatment of migraine (reviewed by Chiechio et al., [35]). Hence, the increase in XA levels we have found in CM patients may represent a compensatory event aimed at reinforcing endogenous analgesic mechanisms in migraine. In this particular case, the high Glu levels may not compete with XA because mGlu2 receptors are preferentially localized in the pre-terminal region of nerve terminals, and are therefore not accessible to synaptically released Glu (reviewed by Nicoletti et al., [36]).

Finally, the functional significance of the large increase in ANA levels we have seen in CM patients is obscure. No specific function or receptor targets for ANA have been described to date. Our findings strongly encourage the study of ANA in models of pain in general and of migraine in particular.

## Conclusions

In conclusion, we have demonstrated for the first time that CM is associated with abnormalities of the kynurenine pathway metabolites. In particular, we found a reduction of KYNA levels with a simultaneous reduction in QUINA levels and increase in XA and ANA levels. KYNA reduction and QUINA increase are consistent with the hypothesis that NMDA receptors are overactive in migraine, and the increase in XA levels may represent a compensatory event aimed at reinforcing endogenous analgesic mechanisms in migraine. Since ANA specific functions or targets are unknown, the significance of its large increase in CM is obscure and these findings strongly encourage the study of ANA in models of migraine.
